# Proposed Neuroimmune Roles of Dimethyl Fumarate, Bupropion, *S*-Adenosylmethionine, and Vitamin D_3_ in Affording a Chronically Ill Patient Sustained Relief from Inflammation and Major Depression

**DOI:** 10.3390/brainsci10090600

**Published:** 2020-08-31

**Authors:** Navzer D. Sachinvala, Naozumi Teramoto, Angeline Stergiou

**Affiliations:** 1 Retired, USDA-ARS, New Orleans, Home 2261 Brighton Place, Harvey, LA 70058, USA; 2 Department of Applied Chemistry, Faculty of Engineering, Chiba Institute of Technology, 2-17-1, Tsudanuma, Narashino, Chiba 275-0016, Japan; teramoto.naozumi@it-chiba.ac.jp; 3 Department of Medicine, Fairfield Medical Center, 401 North Ewing, Lancaster, OH 43130, USA; drangelinestergiou@gmail.com

**Keywords:** multiple sclerosis, major depression, inflammation, dimethyl fumarate, bupropion, S-adenosylmethionine, vitamin D_3_, neuroimmune mechanisms

## Abstract

We had discussed earlier that, after most of the primary author’s multiple sclerosis (MS) symptoms were lessened by prior neuroimmune therapies, use of dimethyl fumarate (DMF) gradually subdued his asthma and urticaria symptoms, as well as his MS-related intercostal cramping; and bupropion supplemented with *S*-adenosylmethionine (SAMe) and vitamin D_3_ (vit-D3) helped remit major depression (MD). Furthermore, the same cocktail (bupropion plus supplements), along with previously discussed routines (yoga, meditation, physical exercises, and timely use of medications for other illnesses), continued to subdue MD during new difficulties with craniopharyngioma, which caused bitemporal vision loss; sphenoid sinus infections, which caused cranial nerve-VI (CN6) palsy and diplopia; and through their treatments. Impressed with the benefit the four compounds provided, in this manuscript, we focus on explaining current neuroimmune literature proposals on how: (1) DMF impedes inflammation, oxidative stress, and cell death in CNS and peripheral tissues; (2) Bupropion curbs anxiety, MD, and enhances alertness, libido, and moods; (3) SAMe silences oxidative stress and depression by multiple mechanisms; and (4) Vit-D3 helps brain development and functioning and subdues inflammation. We realize that herein we have reviewed proposed mechanisms of remedies we discovered by literature searches and physician assisted auto-experimentation; and our methods might not work with other patients. We present our experiences so readers are heartened to reflect upon their own observations in peer-reviewed forums and make available a wide body of information for the chronically ill and their physicians to benefit from.

## 1. Introduction

Recently, we discussed how a male patient (primary author) has battled major depression (MD) since childhood due to amblyopia, dyslexia, asthma, urticaria, and infections that take weeks/months to resolve with antibiotics [1,2]. At 19, he was diagnosed selective immunoglobulin-M (IgM) deficient [3], and, by his mid-40s, required surgeries to remove: his uvula, nasal polyps, nasal cartilage defects, and premature cataracts. Furthermore, when intravenous antibiotics were not used in some surgeries, he suffered infections that took months to heal. The patient belongs to a closed Indo-Iranian minority, Zoroastrians, wherein some consanguineous relations have produced children with neuroimmune conditions [4,5,6]. However, he grew up in and has a very stable family, is well educated, and enjoyed a productive career as scientist [7]. In 2005, he was diagnosed with fulminant MS, which left him mobility impaired, severely depressed, and unable to stand, balance, and coordinate his hands. Three years later, he accepted disability retirement as a research chemist, and his Beck depression inventory (BDI) scores during early years with MS hovered in the 30s to 40s, indicating severe depression [1,2].

Soon after MS was diagnosed, the patient received guidance from his former U. Tehran professors, to note the physiological and psychological changes he experienced, and become informed about his conditions through CME and university courses, as well as current texts and journals. Furthermore, a former neuroscience teacher trained him to: randomly record BDI scores (several times a year), have consistent routines, practice yoga and self-hypnosis (to manage pain, anger, rumination, and sleep), and use diaphragmatic breathing and over the counter NSAIDs to control intercostal and cardiac sphincter cramps [8,9,10,11,12].

For MS between 2005–2014, the patient received: multiple plasmaphereses, mitoxantrone (Novantrone^®^), 6-methylprednasolone, interferon-β-1-α (Rebif^®^), natalizumab (Tysabri^®^), and glatiramer acetate (Copaxone^®^). However, each MS treatment resulted in some gradual improvement with side effects that required its discontinuation. Later, in mid-2014, he was prescribed DMF (Tecfedera^®^), which in addition to reducing MS-related cramps also relieved his asthma and urticaria symptoms. Within a year, DMF induced leukopenia and was discontinued [1,2,13,14].

Likewise, for MD, years of literature study with physician guided trial and error enabled the discovery of Bupropion, which with dietary supplements SAMe and vit-D3, further reduced inflammation, improved everyday functioning, enhanced libido, and enabled euthymia that lasts to date [15,16,17]. Between October 2015 to April 2017, the patient’s BDI scores hovered between 5 and 16, and he enjoyed a pleasant productive interlude with mild mood disturbances related to life-flow events [1,2].

In late April 2017, the patient complained of heat intolerance, nausea, headaches, disorientation, blurry vision and falling, and his MRI’s showed a conjoined (adamantinous and papillary) perisellar tumor, ~0.5 cm^3^, that was diagnosed as craniopharyngioma by imaging and spectroscopy [1,2,18,19]. By year’s end (late November 2017):

- His papillary and adamantinous growths measured 2.3 cm^3^ and 0.7 cm^3^, respectively;
- His visual acuities deteriorated from 20/60 the year before, to 20/100 (left); and 20/25 in 2016 to 20/80 (right);
- He suffered bitemporal vision loss; and
- His coherence tomography data showed compressive atrophy of the left optic nerve [20];
- However, serum levels of his hypothalamic, pituitary, adrenal (HPA) axis hormones from 2016 through 2018 were normal; and his MRIs showed no new MS lesions [1,2,18,19,20,21,22,23].

Furthermore, because the patient has extant immune problems (infections that take a long time to heal, MS, allergies, EDS, and asthma), his tumor was ablated with 30 rounds of fractionated stereotactic radiation treatments (FSRT), and surgery was avoided [1,2,18,19,20,21,22,23].

Three months after his radiation treatments had ended (March 2018), his ablated tumor residue measured ~0.41 cm^3^; his RS visual acuities were 20/25 and 20/70, respectively; his bitemporal vision was almost restored; however, to date, he has central scotomas in both eyes, and compressive atrophy of the left optic nerve, which could be due to chronic type 2 diabetes, and/or MS [1,2].

Thereafter, following a brief pleasant interlude (November 2018 to March 2019), the patient experienced pain in his R/L sphenoid sinuses that upon intensification induced diplopia. Personal observations (colored viscous nasal lavage), and ophthalmic and MRI data all indicated that pain and diplopia could be attributed to sphenoid sinus infections and raised intracranial pressure. This was supported by the observation that use of antibiotics eliminated infection, pain, pressure, and diplopia, and MS as a probable cause of diplopia was eliminated [2]. At present, continuing with the above cocktail and all other routines and medications, his BDI scores are between 6 and 15, he walks a mile in 19–22 min with crutches, swims, scuba dives, runs on-spot multiple times a day with support to control narcolepsy, and participates in writing and editing scientific articles and reviews with his former colleagues [1,2,7,8,9,10,11,12,18,19,20,21,22,23].

Furthermore, it is important to mention that this patient continues to benefit from yoga, meditation, and visualization techniques. However, other than citing the references he uses, none of the authors can authoritatively comment on how or why these methods work [8,9,10,11].

As summarized above, in references [1,2], we showed the years of effort it took to discover DMF, which after all MS treatments were exhausted, finally subdued MS related intercostal cramping and other inflammatory conditions (asthma and urticaria). For this patient, as pain and disability are reduced, disposition improves. However, it took many more years of physician guided trial and error to discover bupropion, and some more years to discover supplements SAMe and vit-D3, which aided the action of bupropion. Therefore, hereafter, we individually discuss current neuroimmune literature that was studied on the four agents for MS and MD to write this review. This is because we want to show that, for this patient, DMF was a very helpful antiinflammatory agent; and use of the bupropion, SAMe, and vit-D3 cocktail in addition to his routines for medication, yoga, and exercises, sustained euthymia and productivity even during disruptions due to craniopharyngioma and abducens nerve (CN6) palsy. We hope that his antidepressant regimen will continue to help him beyond the present day as new challenges arise [1,2,8,9,10,11,12,15,16,17,18,19,20,21,22,23].

## 2. Mini Reviews on the Roles of DMF, Bupropion, SAMe, and Vit-D3

### 2.1. The Neuroimmune Roles of DMF in Subduing Inflammation in the CNS and the Body

Inflammation is a physiological response to injury caused by infections, blunt or sharp trauma (falling or cutting), systemic disease (allergies, asthma, or diabetes), and/or social stress (poverty, divorce, or job loss). Prolonged inflammation results in tissue damage, which in turn has physical and psychological consequences. Physical effects of injury are: pain (caused by release of histamine and bradykinin by injured tissues that stimulate nociceptors in and around damaged tissues); redness, swelling and warmth due to infiltration of blood and lymph at the site of injury; and difficulty in using the injured body part due to pain and loss of function. Psychological effects of injury are anger, helplessness, and depression due to pain and loss. Thus, concurrent to explaining how DMF subdues inflammation, herein we ease the reader into contemporary literature on the mechanisms of inflammation so the reader can appreciate the neuroimmune pharmacology of DMF, bupropion, supplements SAMe, and vit-D3 [12]. Discussion on DMF is divided into the following subsections:

2.1.1. What is DMF and what does it do?
2.1.2. DMF metabolism, and its conjugation with glutathione and nuclear factors NFκB, and NRF2 to suppress inflammation.
2.1.3. Stresses and inflammation.
2.1.4. Effect of inflammation on cells and tissues.
2.1.5. Remarks on DMF pertinent to this case.

#### 2.1.1. What Is DMF, and What Does It Do?

**Figure 1:**
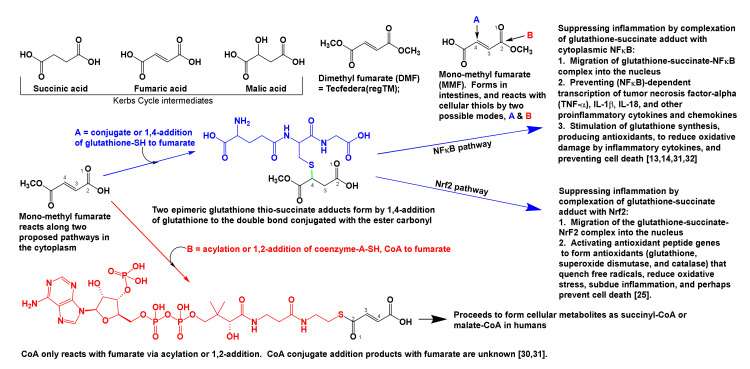
Fumarates and proposed immune modulation mechanisms. Fumaric acid is a Krebs cycle intermediate, wherein, succinic and malic acids are precursor and product, respectively. DMF forms monomethyl fumarate, which crosses the BBB, and is speculated to subdue inflammation by two mechanisms: (1) NFκB pathway: When glutathione thio-succinate adduct complexes NFκB, it is expected to block the synthesis of TNF-α and other proinflammatory cytokines and prevents cell apoptosis, and (2) NrF2 pathway: When glutathione thio-succinate adduct complexes NrF2 it suppresses inflammatory cytokine syntheses and stimulates the production of antioxidants (catalase, glutathione, and superoxide dismutase, etc.) which then quench reactive oxygen and nitrogen free radicals and reduce oxidative stress. Prolonged use of DMF causes leukocytopenia. As opposed to wide spread immune suppression, as seen with steroids, immune modulation is considered targeted therapy because it acts on few physiological defenses. Coenzyme-A adds to fumarate by 1,2-addition (acylation, reaction B) and not by conjugate, 1,4-addition (reaction A). The resulting fumarate-, malate-, or succinyl-CoA adducts form normal Krebs cycle metabolites [13,14,24,25,26,27,28,29,30,31,32].

Fumaric acid is a Krebs cycle intermediate that is formed by dehydrogenation of succinic acid as the cycle continues in mitochondria (Figure 1). Its dimethyl derivative, DMF, is an immune modulator that is currently approved by FDA to treat inflammatory conditions like psoriasis and multiple sclerosis. DMF was introduced to this patient in 2014 after he had completed many immune suppression and modulation treatments between 2005 and 2014. However, DMF was stopped a year later due to leukocytopenia. After several weeks of DMF use, the patient noticed that his MS-related intercostal cramping, and frequent asthma and urticaria symptoms had reduced, which improved his disposition and concentration. About a month thereafter, intercostal cramping stopped [1,2,13,14].

Immune modulators like DMF are reported to reduce concentrations of proinflammatory cytokines, cytotoxic CD8^+^ T-cells, natural killer T-cells (NKT), and T-helper-1 and 17 (Th1 and Th17) cells, and shift innate immune mechanisms to milder, antibody, B- and T-helper-2 (Th2) cell driven adaptive responses. Immune modulation is narrower in scope, as exemplified by omalizumab, Xolair^®^, which blocks the attachment and crosslinking of the immunoglobulin-E-receptor (Fc-epsilon-RI) on mast cells and eosinophils to stop asthma attacks and subsequent destruction of airway tissues [24,25].

In comparison, immune suppressors (e.g., cortisone, prednisolone, and FK-506) shutdown or weaken the immune response. They are speculated to act on aryl hydrocarbon receptors that bind nuclear DNA and generate proteins which regulate mitochondrial cytochrome P450 enzymes. These enzymes metabolize and eliminate foreign molecules. As seen in transplantation cases, immune suppression reduces the efficacy of innate immunity and renders the patient susceptible to opportunistic infections [26,27,28,29].

#### 2.1.2. DMF Metabolism, and Its Conjugation with Glutathione and Nuclear Factors NFκB, and Nrf2 to Suppress Inflammation

DMF is hydrolyzed to monomethyl fumarate in the small intestine by esterases and pH 6–7.4 (Figure 1). Fumaric acid, and mono and dimethyl fumarates readily diffuse into cells and across the BBB due to their lipophilicity and small size (116 to 140 Daltons) [13,14]. In the cytoplasm, sulfhydryl (=thiol = SH) groups of intracellular molecules like glutathione and coenzyme-A add to fumarates. Glutathione adds via conjugate, 1,4-addition, and coenzyme-A (CoA) reacts with fumarates only via acylation (or 1,2-addition), see reactions marked A and B, Figure 1. Thereafter, fumarate-CoA-adducts form Krebs cycle metabolites in cells, however, formation and fate of conjugate 1,4-addition products of CoA with fumarates are unknown in animal biochemistry literature [30,31].

To reduce inflammation, the glutathione-succinate conjugate addition product (reaction A, Figure 1) is suggested to act upon two cytoplasmic signaling proteins: nuclear factor kappa B (NFκB), and nuclear erythroid 2-related factor 2 (Nrf2). The glutathione-succinate-NFκB-complex stimulates cells directly to produce more antioxidants (glutathione and catalase), and prevents passage of the complex into the nucleus, thereby arresting NFκB-dependent transcription of proinflammatory cytokine genes (e.g., IL-1β and IL-18) in activated innate immune cells (dendritic cells, macrophages, and endothelial cells), which otherwise would occur if the glutathione-succinate adduct and NFκB are uncomplexed [24,25]. Production of antioxidants and reduction of proinflammatory cytokine concentrations in cells is antiinflammatory and it prevents cell death. Likewise, cytosolic concentrations of chemokines (CCL2, and CXCL10) are also reduced when the glutathione–succinate–NFκB complex does not interact with nuclear chromatin.

IL-1β, among its many functions, helps affect systemic and local inflammation in response to stress, infection, and/or injury. It induces fever, activates lymphocytes, and promotes their infiltration into sites of infection or injury [32]. Likewise, IL-18 among its many immune functions helps T-helper-1 (Th1) cells activate cytotoxic T-cells, which in turn participate in the destruction of injured or infected cells [33]. Both interleukins have other functions that are beyond the scope of this brief discussion. In addition, while pro-inflammatory cytokines activate innate immune cells to destroy infected or damaged cells, chemokines (which are also low molecular weight cytokines) signal innate immune cells to migrate from the tissues in which they reside to sites of inflammation [13,14,31,32].

Many genes possess an NFκB binding component, and it is yet unknown if the glutathione-succinate-NFκB binds all NFκB-related histones and genes, or only complexes specific chromatin regions. Furthermore, the addition of glutathione to fumaric acid produces two epimeric products (shown by a green-colored wavy sulfur–carbon bond in Figure 1), and whether a single diastereoisomer or both glutathione–succinate adducts silence proinflammatory cytokines, and stimulate the production of antioxidants are unknown [13,14,24,25,30,31,32,33,34,35].

Upon combining with cytoplasmic Nrf2 protein, the glutathione-thiosuccinate-Nrf2 complex is expected to migrate into the nucleus, activate the antioxidant defense system genes, and generate antioxidants (glutathione, superoxide dismutase, and catalase) that quench free radicals, avert oxidative stress, subdue inflammation, and preclude NLRP3 inflammasome mediated pyroptosis [24,25,30,31,32,33,34,35].

#### 2.1.3. Stresses and Inflammation

Pathogen associated molecular patterns (PAMPs) and cellular damage associated molecular patterns (DAMPs) are recognized by their matching pattern recognition receptors (PRRs) that reside in immune cells, and these complexes cause inflammation in the CNS and periphery. Inflammation due to both PRR-DAMP and PRR-PAMP-complexes, results in the release of the same inflammatory cytokines and chemokines that occurs upon inflammasome activation. Inflammasome activation can occur with infections, systemic disorders (like asthma or diabetes), or psychogenic stress (e.g., job loss, death in family). Inflammasomes are cytosolic multimeric protein complexes that innately recognize these dangers (cellular stresses, oxidative damage, DAMPs and/or PAMPs) and activate cellular machinery to produce inflammatory cytokines. Therefore, inflammation is triggered when cellular machinery recognizes dangers, viz, PRRs complexes of PAMPs or DAMPs [35,36].

Receptor binding sites (also called ligands) that indicate cellular stress and tissue damage are called DAMPs. DAMPs are endogenous molecules that are either amplified within cells, or are spilled extracellularly after cells experience stress, or they die by necrosis or pyroptosis, but not apoptosis (which is natural cell death). Sterile inflammation occurs with psychogenic stress, and/or autoinflammation, and these DAMPs bind PRRs. Some examples of DAMPs of sterile inflammatory conditions are extracellularly spilled adenosine triphosphate (ATP), heat-shock protein 72 (Hsp72), and uric acid crystals (along with other cellular contents) that are also spilled in infections and metabolic diseases [35,36].

The transcription factor NFκB can directly activate macrophages to produce IL-1β and IL-18. However, binding of DMF with glutathione and then with NFκB arrests NFκB mediated inflammation [37]. The transcription factor NRF2 activates the NLRP3 inflammasome in two steps and triggers production of the same inflammatory cytokines. However, formation of the DMF-glutathione-NRF2 complex results in antioxidants that attenuate the effects of IL-1β and IL-18, to prevent inflammation, and cell death (Figure 1) [38]. Priming is the first step in activating the inflammasome, i.e., PRRs recognize danger and recruit cellular machinery to form NLRP3. Thereafter, if PRRs bind PAMPs and/or DAMPs, for a second time, they trigger cellular (e.g., macrophage) machinery to build more inflammasomes in the cytoplasm, and activate proinflammatory caspases (cysteine-aspartic proteases) to cleave proinflammatory cytokines (pro-IL-1 or pro-IL-18), to form mature IL-1α or β or IL-18, and release them from the cell [35,36,37,38,39].

Inflammasomes comprise a cytosolic sensor of danger, which is a nucleotide binding domain, an adaptor protein called apoptosis-associated speck-like protein containing a caspase recruitment domain (ASC), and an effector caspase (caspase-1). In addition, NLR stands for nucleotide-binding-oligomerization domain with leucine rich repeating units that collectively sense danger. The acronym P3 in NLRP3 stands for a protein that can bind the intracellular protein ubiquitin to regulate NLRP3 activity but not destroy the inflammasome. What triggers this binding and activation of ubiquitin to regulate inflammasome activity is at present unknown. Out of the many known inflammasomes, the NLRP3 inflammasome recognizes inflammation provoked by PAMPs and DAMPs in the CNS and the periphery, and is implicated in many inflammatory diseases including MS and MD [36,40].

Inflammation in the central nervous system (CNS) affects brain functions as well as moods, from mania to severe MD, and MD or MS sufferers for example, show elevated concentrations of inflammatory molecules (IL-1β, IL-2, IL-6, IL-18, TNF-α, nitric oxide, IFN-γ, etc.) in sera and CSF. Their frequency of appearance in serum and CSF shows disease recurrence, and concentration increases indicate enhanced severity of disease. Repeated severe stress brought on suddenly, in the absence of physical injury, pathogenic disease, or encounter with a toxin causes emotional dysregulation as well as increases inflammatory substances in tissues and blood in animals and humans. This is called sterile inflammation. Sterile stress can be psychological or systemic and occurs in the absence of overt tissue damage by trauma, toxin, or microbe, and it provokes the same kind of systemic and CNS inflammation, detrimental to physical and mental health. Just as is seen in the sera of patients following toxin or microbial invasion, exposure to acute or repeated life stressors (dealing with chronic illness, job loss, divorce, death of spouse, threat, isolation, rejection, as well as engaging in very strenuous physical exercises) increases the same inflammatory markers, viz, C-reactive protein (CRP), interleukins (IL-1, IL-6, IL-18, etc.), soluble tumor necrosis factor-α receptor (TNF-α), and NFκB mRNA (inflammatory proteins transcription factor) in leukocytes, which cause mood disorders [41,42,43,44,45,46].

The NLRP3 inflammasome is implicated in many sterile inflammatory diseases including ischemia-reperfusion injury, autoinflammatory diseases, type 2 diabetes, gout, obesity, atherosclerosis, and Alzheimer’s disease, and these sterile inflammatory diseases often cooccur with MD. In addition, there is clinical evidence that patients with MD have increased concentrations of NLRP3 and caspase-1 in their peripheral blood mononuclear cells (DCs, macrophages, etc.) [46,47,48].

#### 2.1.4. Effect of Inflammation on Cells and Tissues

Inflammation causes destruction of both damaged or infected cells as well as neighboring tissues before repairs can occur. In chronic inflammation, damage to cells, tissues, and organs is prolonged, which leads to other diseases; e.g., type-2 diabetes, cancers and atherosclerosis [41,42,43,44]. In MD, MS, and other inflammatory diseases, the cytokines, which signal the presence of danger, such as exogenic stresses, foreign agents (pathogens or toxic molecules), are interleukins: IL-1, IL-2, IL-6, IL-18, TNF-α, as well as IFN-γ. These are produced in patients by CNS microglia, and macrophages, dendritic cells, B-cells, and CD4^+^ T-helper cells (Th1 and Th17), which infiltrate the CNS to fight the source of pathology [36,37,45,46,47,48]. Peripheral blood mononuclear cells of stressed animals and humans enter the CNS via the BBB, the circumventricular organs, or by binding receptors on brain endothelial cells that transduce them into the brain. Alternatively, cytokines, PAMPs, and DAMPs activate afferent vagal nerve branches that in turn activate the limbic system and the anterior cingulate and dorsolateral prefrontal cortices [36,37,39,40,43,45,46,47,48].

In chronic inflammatory illnesses like MS and MD, CNS tissue losses are seen in the anterior and subgenual cingulate cortices, amygdala, prefrontal and orbitofrontal cortices, the ventral striatum, and the hippocampus [49]. The lesion burden in the hippocampus and the temporal lobes are proposed to indicate severity of MD. Moreover, because the hypothalamic, pituitary, and adrenal (HPA) axis is very active in inflammation and MD, the pituitary gland region may be enlarged in tomographic and magnetic resonance images of some patients. Consequently, in these situations, fatigue and cognitive decline become co-occurrences. Fatigue complaints can be correlated with atrophy of cortical gray matter, basal ganglia, and the frontal lobe. Reduced attention is matched with lesions in the parietal lobes. Anatomic signs that portend cognitive decline include: third ventricle enlargement; atrophy of cortical gray matter, hippocampi, thalamic nuclei, thalamocortical tracts; and the overall lesion burden on the patients appear in their CNS MRIs. In addition, many chronically depressed patients show occipital lobe bending for reasons not yet explained in the literature [48,49,50,51,52].

Collective involvement of vagus nerve fibers (as seen by transcranial stimulation treatments), and immune, hypothalamic, pituitary, and adrenal (HPA) axes of depressed patients, alters glucose, lipid, tryptophan, and other neurotransmitter metabolisms. These changes are associated with elevations in serum cortisol level, which indicate abnormal carbohydrate and lipid metabolism; and decreased brain derived neurotrophic factor (BDNF), which in turn indicates poor neuronal health. Imbalanced glucose utilization, can be seen in positron emission tomographic (PET) images by:

- Decreased accumulation of isotopic ^18^F-2-fluoro-2-deoxyglucose (2FDG), which indicates below normal energy metabolism (or glucose use) in the bilateral insula, left putamen, left globus pallidus, right caudate nucleus, and the cingulate gyrus, and specifies inflammation, major depression, and cognitive decline; and
- Increased accumulation of 2FDG in the thalamic nuclei and hypothalamus indicates, increased energy metabolism, aging, and emotional agitation [38,39,40,41,42,43,44,52,53,54,55,56].

Furthermore, in patients with MS and MD, abnormal glucose and fatty acid metabolisms are associated with increased β-hydroxybutyrate, acetoacetate, and acetone, which indicate likely obesity and type-2-diabetes as comorbidities [55,56].

**Figure 2:**
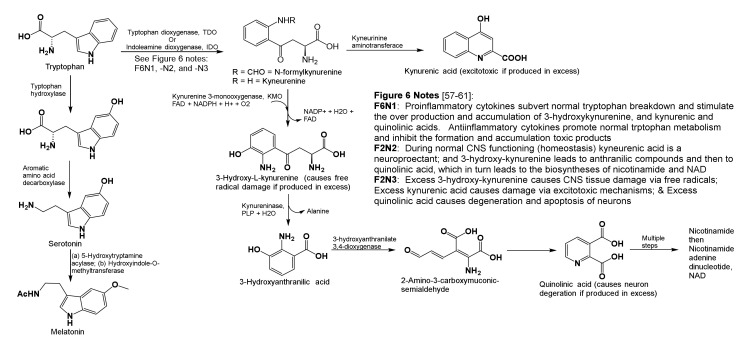
All tryptophan metabolites are normally produced in the CNS. In chronic stress (psychogenic or in diseases), proinflammatory cytokines help over-produce kynurenic acid, 3-hydroxykynurenine, and quinolinic acid, their excess accumulation in CNS causes oxidative damage, excitotoxicity, and neuron loss [57,58,59,60].

Altered tryptophan metabolism is seen in MD (Figure 2), which causes decreased serum tryptophan levels, increased indole-2,3-dioxygenase activity, overproduction of 3-hydroxykynurenine and kynurenic and quinolinic acids. Their accumulation in the CNS adds to tissue damage. During homeostasis, 3-hydroxykynurenine is a neuroprotectant that filters UV light and prevents cataract formation. However, during inflammation, its over production and accumulation promotes formation of oxidative free radicals that cause tissue damage, neural cell apoptosis, and scar formation. Likewise, during homeostasis, kynurenic acid is a neuroprotectant that helps normal glutamate, *N*-methyl-D-aspartate (NMDA) and α-amino-3-hydroxy-5-methyl-4-isoxazolepropionic acid (AMPA) receptor functioning. When present in excess, kynurenic acid causes excitotoxicity. In psychologically stressful situations, chronic diseases, and MD, proinflammatory cytokines encourage overproduction of glutamate by excessively exciting NMDA receptors. This is called excitotoxicity. It causes neuron death by an overflow of glutamate, aspartate, and calcium ions from receptors at synapses (Figure 2). Similarly, during homeostasis, cells produce limited quantities of quinolinic acid en route to making the important coenzyme nicotinamide adenine dinucleotide (NAD^+^), which is needed in cellular redox reactions. In stress and diseases, inflammatory cytokines produced by activated microglia and macrophages overproduce quinolinic acid instead of NAD^+^, and accumulation of quinolinic acid in neural tissues leads to neurodegeneration and apoptosis (Figure 2) [57,58,59,60].

#### 2.1.5. Remarks on DMF Pertinent to This Case

Regardless of the precise mechanisms by which glutathione-thiosuccinate-complexes reduce inflammation and tissue damage (Figure 1 and Figure 2), the long-term side effect of high dose immune modulation by fumarate is the depletion of available sulfhydryl groups (thiols, R-SH) needed for cellular redox reactions, and for T-cell receptor functioning. Irreversible binding of thiols of immune cell receptors via conjugate addition to fumarate are reported to induce apoptosis and reduce T-cell populations. Furthermore, the same conjugate or Michael addition mechanism (reaction A in Figure 1) also forms the basis for how some anticancer drugs are designed to inhibit spindle fiber formation. At present, there are no reports in the literature on DMF-mediated exacerbation of major depression in MS, psoriasis, or other conditions treated with this immune modulator [60,61].

### 2.2. Proposed Neurochemical Roles of Bupropion in Subduing Inflammation and MD

Discussions on bupropion involve the following subsections:

2.2.1. Bupropion structure, metabolic activation, and what does the drug do?
2.2.2. Value added advantages of using bupropion
2.2.3. Remarks on bupropion pertinent to this case

#### 2.2.1. Bupropion Structure, Metabolic Activation, and What Does the Drug Do?

**Figure 3:**
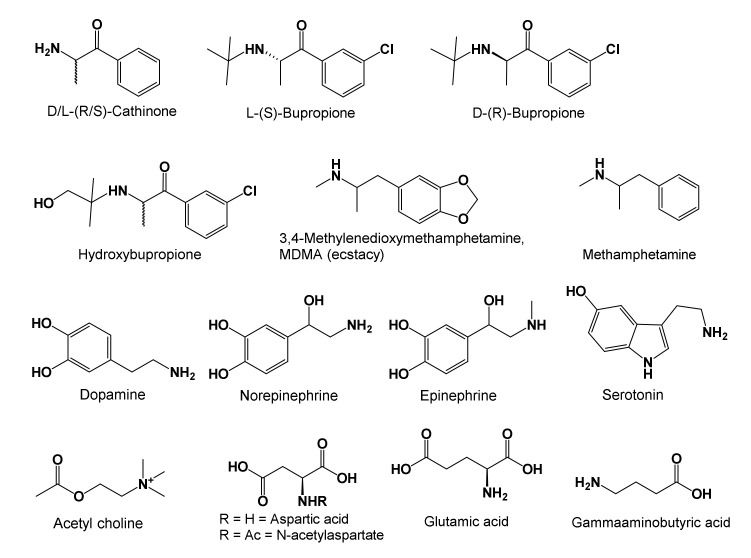
Structures of cathinone, bupropions, amphetamines, and of neurotransmitters as discussed in the text and in Box 1 [63,64,65,66,67,68,69,70,71].

Bupropion is a cathinone derivative with a *t*-butyl group attached to the amine nitrogen, and a meta-chloro-substituent on its phenyl ring (Figure 3) [62]. Bupropion in tablet form is sold as a racemic mixture of D(R) and L(S) isomers, and 6-hydroxybupropion is its metabolite formed by action of cytochrome P450-B26 in hepatic mitochondria. After bupropion is consumed, it metabolizes to hydroxybupropion, which survives in the serum at least 10 times longer than the parent drug. It is a stimulant and antidepressant that selectively binds norepinephrine receptors [63,64,65]. For readers’ convenience, Figure 3 shows structures, and Box 1 summarizes CNS and PNS roles of low molecular weight neurotransmitters relevant to our discussion (summaries in Box 1 were prepared from this text and cited references). Pharmacology of bupropion resembles that of cathinone, a stimulant isolated from the leaves of the khat plant (*Catha edulis*). Cathinone, bupropion, methamphetamine, and ecstasy (3,4-methylenedioxymethamphetamine) are alike in their structures (Figure 3), pharmacology, and potential for abuse (because they can be hallucinogenic when misused). Consequently, they are controlled substances in the US and other nations [65]. Regardless of its legal status, the chewing of khat plant leaves is pervasive in the Middle-East, North and Central Africa, and the Indian Subcontinent [65]. Bupropion readily diffuses into cells and across the BBB due to its small size and lipophilicity. Unlike most SSRIs and SNRIs, as well as tricyclic antidepressants (TCAs) that induce erectile dysfunction, weight gain, and added depression in patients, bupropion and its metabolites do not impart such side effects [62,63,64,66]. In addition, some SSRIs, SNRIs, and TCAs increase the concentration of the inhibitory neurotransmitter **γ**-aminobutyric acid, GABA, and add to depressive symptoms in a few patients. Bupropion does not act upon GABA receptors in the CNS. However, it can cause disinhibition and may not be prescribed to schizophrenia and bipolar disorder patients who experience manic and depressive episodes [66,67,68]. Furthermore, bupropion mildly stimulates nitric oxide secretion needed to maintain an erection, and sustains adequate serotonin levels in the body to facilitate orgasms (Figure 3 and Box 1) [69,70,71].

As a stimulant, bupropion blocks dopamine and norepinephrine transporters to suppress their reuptake and maintain adequate concentrations at synapses (Box 1). Likewise, bupropion weakly suppresses serotonin reuptake from synaptic spaces as well. Maintaining adequate dopamine, serotonin, and norepinephrine neurotransmitter concentrations in CNS is proposed to sustain equanimity per the monoamine hypothesis of MD [69,70,71].

#### 2.2.2. Value Added Advantages of Using Bupropion

A value-added advantage was discovered when bupropion, 300 mg was supplied to Scandinavian athletes the evening before and the morning of their experimental endurance trial, all athletes showed improved performance in 30 °C (86 °F), and 48% relative humidity environment. It was proposed that the drug acted on the adrenalin system (Box 1), without increasing perceptions of exertion or heat exhaustion [72].

Furthermore, dopamine depletion in tobacco smokers is proposed to cause cravings and addiction behaviors. Bupropion and hydroxybupropion were found to reduce cravings to smoke tobacco by blocking dopamine and norepinephrine reuptake and by keeping their optimal concentrations in reward and alertness centers of the brain [62,63,66,68].

Box 1 **Low MW neurotransmitters, ability to cross BBB, production, and PNS and CNS effects.**

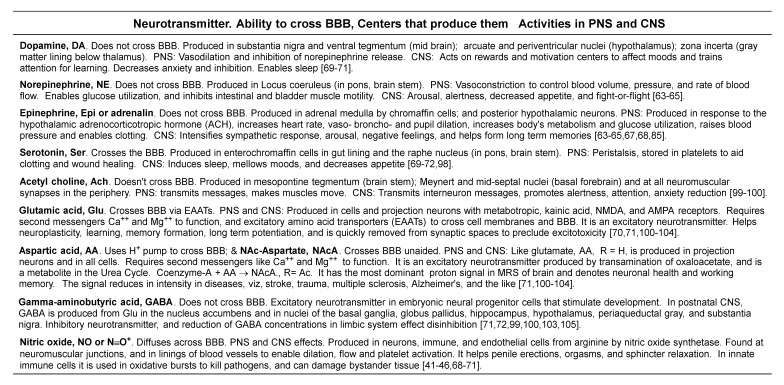

Dopamine is released from substantia nigra and ventral tegmentum of the midbrain, and nucleus accumbens in the basal forebrain. It focuses attention when learning, induces feelings of being empowered and rewarded, and is also implicated in addiction behaviors. Dopamine is a precursor to norepinephrine that is synthesized in the locus coeruleus. In addition, serotonin is synthesized from tryptophan in the raphe nucleus (Box 1). Both the locus coeruleus and the raphe nuclei are clusters of neurons in the midbrain, pons, and medulla (collectively recognized as regions of the brainstem) [71,72,73,74,75,76,77].

In the CNS, serotonin stabilizes moods, affords feelings of satiety, induces sleep, and helps with learning and memory formation. In the periphery, serotonin is stored in blood platelets, and is made in the neuromuscular junctions of smooth muscles lining blood vessels and intestines. Here, serotonin helps with blood coagulation, wound healing, and smooth muscle contraction. In the CNS, norepinephrine enhances alertness and learning. In stressful situations, norepinephrine induces urges to either fight threat or flee from it. In the spinal cord, norepinephrine enhances the perception of pain. In the periphery norepinephrine and its receptors serve multiple roles in: energy metabolism, blood pressure regulation, immunity, and enabling the kidneys, heart, viscera, entrails, and pancreas to function [70,71,72,73,74,75,76,77].

In addition, bupropion antagonizes nicotinic acetylcholine receptors (NAChR) in the autonomic nervous system and at neuromuscular junctions. This valuable property of NAChR antagonism relieves airway constriction in sleep apnea and asthma [76]. In the CNS, acetylcholine transmits interneuron messages, and plays roles in alertness, attention, and anxiety reduction. In the peripheral system, acetylcholine conducts neuromuscular messages that expedite smooth and skeletal muscle contraction. Administering bupropion to elderly patients reduces acetylcholine deficiency and lessens cognitive decline as well as the symptoms of delirium and dementia [70,71,72,73,74,75,76,77].

In combination with mild exercises, bupropion is used to treat pain in fibromyalgia patients because it suppresses the synthesis of proinflammatory cytokines IFN-γ and TNF-α, and promotes the synthesis of antiinflammatory cytokines IL-2 and IL-10. While IFN-γ drives inflammation and neuropathic pain, TNF-α intensifies them, and both IFN-γ and TNF-α play roles in inflaming MD [66,67,68,78]. When released in regulated amounts (e.g., by dendritic cells), IL2 helps the thymus develop T-cells that are self-tolerant and regulatory. These T-cells suppress action of autoreactive T cells that may attack normal healthy cells and tissues. IL-2 also promotes formation of memory T cells that help the body fight reinfection by the same pathogen. IL-10 is antiinflammatory because it inhibits the syntheses of proinflammatory cytokines (IFN-γ, TNF-α, and granulocyte monocyte colony stimulating factor, GM-CSF), which are proteins made by activated immune cells during inflammation [78,79,80].

#### 2.2.3. Remarks on Bupropion as They Pertain to This Case

It was this knowledge of bupropion’s minimal side effects and anti-inflammatory, pain relieving, muscle relaxing, heat tolerance enhancing, and anti-depressive properties that prompted its use in conjunction with the patient’s regular medications, and above-mentioned routines to gain euthymia [78,79,80]. While the patient has made much progress in reducing inflammation and major depression, despite occasional health-related setbacks, he needs to find ways to decrease his body weight despite his use of steroids for asthma and urticaria, and improve his heat endurance [1,2].

### 2.3. Proposed Neuroimmune Roles of SAMe in Subduing Inflammation and MD

As a co-reactant in biochemistry SAMe is involved in methyl-, sulfur- and amino-propyl groups transfers, and these reactions have far reaching effects in physiology. Thus, discussions herein on SAMe will follow the outline:

2.3.1. SAMe structure, biosynthesis, and stability in aqueous solutions;
2.3.2. Designation, commercial production, formulation, diastereoisomeric purity, and degradation products in water;
2.3.3. Transport and concentration of SAMe in tissues in health and disease
2.3.4. One carbon transfer, transsulfuration, and physiological effects
2.3.5. Other one-carbon cofactors and physiological actions.
2.3.6. Transfer of aminopropyl groups, and physiological effects
2.3.7. Inflammation reduction and remarks on SAMe pertinent to this case

#### 2.3.1. SAMe Structure, Biosynthesis, and Stability in Aqueous Solutions

**Figure 4:**
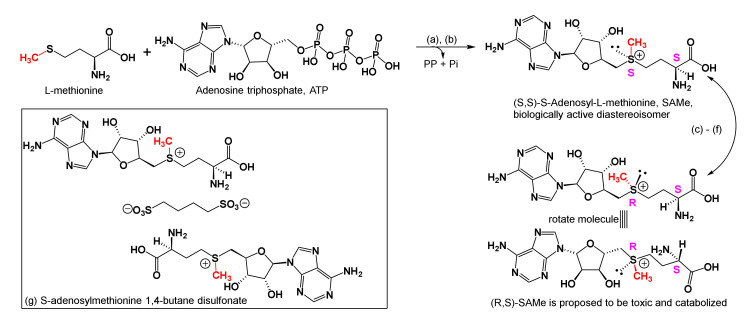
Biosynthesis of SAMe, configurations of biologically active and inactive diastereoisomers, its relative concentrations in serum, and CSF, and a cartoon of the commercial SAMe 1,4-butane disulfonate formulation. (**a**) In all cells, the reaction of ATP, water, and methionine, catalyzed by ATP:methionine adenosyltransferase, MAT, EC 2.5.1.6, is proposed to provide only the S,S-diastereoisomer of SAMe, MW = 398.4 Daltons, plus pyrophosphate, PP (=H_2_P_2_O_7_^2−^), and inorganic phosphate, (Pi = H_2_PO_4_^−^). However, the R,S-diastereoisomer is also found in nature. This reaction is irreversible. (**b**) In the S,S-configuration, substituents about the sulfonium ion (sulfur cation) and the α-amino acid carbon are both counterclockwise and designated S (means left handed or counter-clockwise). In the R,S-diastereoisomer, substituents about the sulfur cation are right-handed, clockwise. (**c**) At pH ~ 1 and −80 °C, SAMe is stable, however, under physiological conditions (pH 7.2 and 37 °C) in vitro, chirality at the sulfur cation racemizes to provide the R,S- plus S,S- diastereomeric mixture, and SAMe decomposes. Post aqueous decomposition, chirality at the methionine α-carbon remains unchanged. (**d**) In the R,S-configuration, SAMe might be toxic, and could be catabolized for use in other physiological cycles; therefore, it is considered biologically inactive. (**e**) For diagnostic purposes, healthy ranges for SAMe in serum are (~30–90 nM), in CSF (~100–420 nM), and lower serum and CSF concentrations of SAMe indicate inflammation. (**f**) Serum levels of SAMe in aggressive and manic patients normally exceed the standard range (see text) and SAMe supplementation for these patients is contraindicated. (**g**) *S*-adenosylmethionine 1,4-butane disulfonate is found in OTC oral supplements [82,83,84,85,86,87,88,89,90,91,92,93,94,95,96].

In all cells, the reaction of methionine, water, and adenosine triphosphate (ATP), catalyzed by methionine adenosyltransferase (MAT, EC 2.5.1.6) produces SAMe (MW 398.4 Daltons), pyrophosphate (PP = H_2_P_2_O_7_^2−^), and inorganic phosphate (Pi = H_2_PO_4_^−^). For the most part, SAMe in all animals is produced and catabolized in the liver (Figure 4) [81,82]. There are two types of MAT: MAT1A is found exclusively in the liver, and MAT2A is found in the fetal liver and post birth in other tissues [83]. Furthermore, while both diastereoisomers of SAMe are possible, the major S,S diastereoisomer is speculated to be biologically active, and the minor R,S diastereoisomer is considered to be inactive because the R,S-adduct could be toxic and might be degraded to its components for reuse in other biochemical pathways [82,83,84]. However, in rat liver transplant experiments, both S,S- and R,S-diastereoisomers of SAMe were shown to react with purine-receptors, and the R,S compound more than its S,S counterpart was shown to increase bile production, and ameliorate ischemia reperfusion injury [85].

#### 2.3.2. Designation, Commercial Production, Formulation, Diastereoisomeric Purity, and Degradation Products in Water

In the literature, *S*-adenosylmethionine is abbreviated as SAMe, SAM-e, SAM, or Adomet, wherein ado stands for adenosine and met for methionine. Since SAMe was frequently seen in articles we studied, we’ve used SAMe throughout this manuscript as well.

SAMe is mass produced via fermentation in *S. cerevisiae* and *E.coli* and is available over the counter in doses (OTC) ranging from 400–1600 mg per capsule [86,87].

It is stable at pH 1 at ca. −80 °C, however, at 37 °C and pH 7.2 (physiological conditions), chirality at sulfur racemizes (forming both diastereoisomers), and SAMe decomposes. OTC SAMe is stabilized as its para-toluenesulfonate (tosylate = *p*-CH_3_-C_6_H_5_-SO_3_^−^) or 1,4-butane disulfonate adducts (Figure 4). Nonetheless, OTC SAMe was shown by proton NMR to contain 5 to 40% of the R,S-diastereoisomer [86,87,88].

In vitro decomposition products of SAMe under physiological conditions are 5-adenosylhomocysteine, adenosine, methanol, adenine, 5-deoxyribosylmethionine, 5′-thiomethyladenosine, 5-thiomethyl-5-deoxy ribose, homoserine and homoserine lactone [81,82,83,84,85,88]. Decompositions of SAMe by reactive oxygen and nitrogen free radicals in innate immune cells were shown to be radical forms of the same structures seen in ionic decompositions, and among them, the 5-deoxyadenosyl radical (Box 2) was prevalent. Endogenous radical processes in physiology are known to occur in mitochondria, peroxisomes, endoplasmic reticulum, and phagolysosomes, and since discussions on radical biochemistry of SAMe exceed the scope of our present review, interested readers are directed to references [82,83,97] and citations therein.

#### 2.3.3. Transport and Concentration of SAMe in Tissues in Health and Disease

SAMe is transported through cell membranes by the soluble ligand carrier family protein member #25 (SLC25), and through the BBB, by the creatine transporter SLC6A8. Creatine is *N*-methylguanidinoacetic acid that forms phosphocreatine, which then helps ATP synthesis and raises cellular energy [89,90].

SAMe concentrations in tissues and blood cells are reported to be in micro-molar (micro-M, μM) ranges, whereas, in serum and CSF, they are nano-molar (nano-M, nM). The standard ranges for SAMe in healthy human serum are ~30–90 nM, and in the CSF ~100–420 nM. Lower than normal SAMe levels are noted in sera of the chronically ill, and in patients susceptible to viral infections, cancer, chronic inflammatory neuroimmune conditions, and those experiencing cognitive decline, e.g., chronic type-2 diabetics and Alzheimer’s patients [91,92,93].

Symptoms of inflammation, pain (osteoarthritis and fibromyalgia patients), vacuolar myelopathy, liver disease, reperfusion injury, and MD were shown to be attenuated with SAMe supplementation with or without use of added NSAIDS or antidepressants. Furthermore, SAMe supplementation at 800 mg/day was reported to significantly lower serum cholesterol in cardiovascular patients (or at risk for it) [89,90,91,92,93,94,95,96].

#### 2.3.4. One Carbon Transfer, Transsulfuration, and Physiological Effects

**Figure 5:**
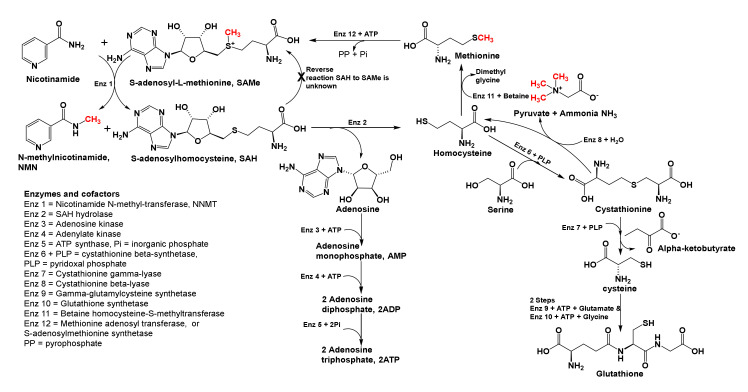
Methyl transfer by SAMe, B_12_, or betaine; syntheses of ATP, cystathionine, cysteine and glutathione; and regeneration of SAMe. Methyl and other alkyl group transferases take the name of the substrate they methylate or alkylate; for example, nicotinamide-*N*-methyl transferase, NNMT methylates nicotinamide, as shown here. All methyl groups in transfer reactions herein are shown in bold red font. SAMe and NNMT convert nicotinamide to NMN and SAH. However, SAH does not directly revert to SAM; instead, SAH is hydrolyzed to adenosine and homocysteine. Either a molecule of SAMe, vitamin B_12_, or betaine can convert homocysteine to methionine, which then reforms SAMe. Upon formation, adenosine is eventually converted to ATP, and homocysteine to cystathionine that transulfurates with serine to form cysteine, and then glutathione [95,96,98,99,100,101,102,103,104,105,106,107,108,109].

SAMe is a ubiquitous cofactor in one carbon metabolism (Box 2). It is a methyl donor, and the byproduct of methyl transfer by SAMe to any substrate is *S*-adenosylhomocysteine (Figure 5). For readers’ convenience, Box 2 shows the cofactors involved in one carbon metabolism and summarizes their suggested roles in subduing inflammation and major depression. To explain methyl transfer, Figure 5 illustrates how SAMe is involved in the syntheses of *N*-methyl-nicotinamide (NMN), ATP, cystathionine, cysteine, and glutathione. As shown therein, SAMe transfers a methyl group to nicotinamide to form NMN, which in turn helps improve cognition, and elevates moods by blocking acetylcholine efflux from cortical, subcortical, limbic, and diencephalic neurons (Box 1 and Box 2). Furthermore, SAMe promotes dopamine norepinephrine, and serotonin secretions that elevate mood as well (Box 1). In patients with major depression, serum tryptophan, serotonin, dopamine, norepinephrine, and brain derived neurotrophic factor (BDNF) levels are reduced, and SAMe supplementation along with physical exercises is reported to help replenish them, increase cerebrovascular perfusion, and afford relief at levels comparable to tricyclic antidepressants [98,99]. However, aggressive, bipolar disorder, and schizophrenia patients who experience mania may not use SAMe because, during mania, serum concentrations of dopamine, norepinephrine, and SAMe are high, and that of GABA, the inhibitory neurotransmitter, is low [99,100]. See Box 1 and Box 2 for convenient summaries on low MW neurotransmitters and cofactors that decrease symptoms of inflammation and MD.

Box 2 **Cofactors in one carbon metabolism, molecular fragments they transfer, and their suggested abilities to reduce inflammation and MD symptoms.**

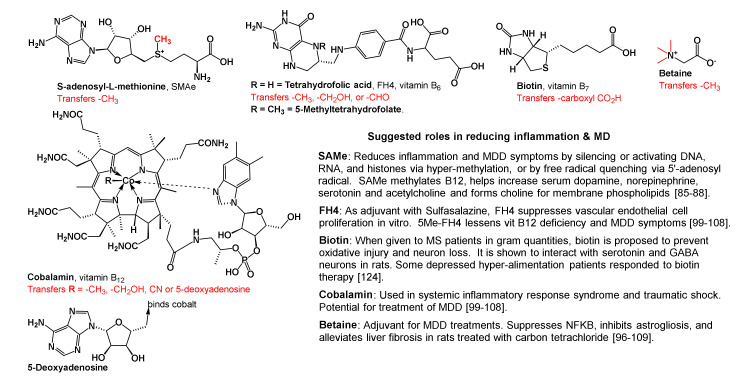

While mechanisms of how SAMe silences or activates genes by methyl transfer to CpG rich promoter regions and/or histones (lysine and arginine residues) are unclear [100], SAMe is proposed to silence RELN production in GABAergic neurons by transferring its methyl group to DNA, RNA, or histones associated with the RELN gene. RELN is an extracellular matrix protein that is proposed to control intercellular connections, as well as neuron migration during fetal brain development. Deficits in RELN are associated with illnesses like schizophrenia, autism, epilepsy, and others. While glutamic and aspartic acids are excitatory neurotransmitters that help long-term potentiation (LTP), GABA is inhibitory that in mature neurons curb impulsive and aggressive behaviors (Box 1) [100,101,102,103,104].

In addition, since glutamate concentrations are high (causes excitotoxicity), and GABA concentrations are low (causes disinhibition) in chronically ill and manic-depressive patients, they are known to exhibit aggressive behaviors due to lack of impulse control. SAMe curbs GABA production by silencing RELN. Thus, with dopamine and glutamic acid levels already high, curbing GABA with SAMe could explain unacceptable behaviors in patients experiencing mania, bipolar disorder, schizophrenia, and chronic pain. Consequently, SAMe supplementation in these patients is contraindicated (Box 1 and Box 2) [99,100,101,102,103,104,105].

#### 2.3.5. Other One-Carbon Cofactors and Physiological Actions

As summarized in Box 2, SAMe is one of five cofactors involved in one carbon metabolism, and each cofactor is expected to reduce inflammation and major depression by a different mechanism. In biological reactions, SAMe transfers a fully reduced carbon cation, (CH_3_^+^, methyl cation) to nucleobases in DNA, RNAs, and terminal nitrogen groups in lysine and arginine residues in histones to silence or enhance gene expression. SAMe also methylates vitamin B_12_ and tetrahydrofolic acid (FH4), to replenish their serum levels (Box 2, Figure 5). FH4 transfers either a formyl (-CH=O) or hydroxymethyl (-CH_2_OH) group to substrate, and its 5-methyl derivative (5-Methyl-FH4) transfers a methyl group to substrate. Thus, following oral consumption of folate, additional amounts of 5-Methyl-FH4 form and cross the BBB to ameliorate vitamin B_12_ deficiency and its associated symptoms, viz, anemia, weakness, depression, and other neurological difficulties. Furthermore, other one carbon metabolism cofactors (Box 2) are reported to lessen inflammation, and disease symptoms in MS, traumatic shock, hepatotoxicity, and MD, to name a few [99,100,101,102,103,104,105,106,107,108].

In biological methylations that employ SAMe (Figure 5), *S*-adenosylhomocysteine (SAH) is the byproduct that is rapidly broken down to homocysteine and adenosine. Furthermore, the direct reverse reaction SAH to SAM is unknown in biochemistry. High serum concentrations of SAH and homocysteine cause oxidative damage to DNA, which is reported to lead to: prenatal and early development maladies; cancers; chronic cardiovascular diseases; and Alzheimer’s disease with age. Later, as shown in Figure 5, adenosine is converted to adenosine triphosphate (ATP, to raise cellular energy), and homocysteine is remethylated by either vitamin B_12_ or betaine to provide methionine, which progresses to restructure SAMe. Alternatively, homocysteine reacts with serine to form cystathionine which forms cysteine via trans-sulfuration, and then glutathione. The latter, glutathione (see Figure 1) is used by cells to quench harmful free radicals and reduce oxidative stress. In addition, the former cysteine stabilizes complex structures of proteins, detoxifies heavy metals, participates in electron transfer reactions, and has other metabolic functions [95,96,98,99,100,101,102,103,104,105,106,107,108,109].

Methylation by SAMs is involved in: gene (DNA and RNA) expression and functioning, clearance of nicotinamide, hormone syntheses, and metabolism and clearance of catecholamine neurotransmitters and other small molecules. Furthermore, while *N*-methylations of lysine and arginine residues in histones, *C*-methylations in genes, and *O*-methylations in RNAs are known to silence or enhance gene expression; hypomethylation of chromatin (DNA, RNA, histone, and related proteins) could either activate or leave gene function (s) unaltered, which in turn, can lead to cancers and/or inflammatory diseases. The exact histones, DNAs, RNAs, and proteins SAMe methylates to afford anti-inflammatory and antidepressant effects are thus far unknown. Activation and/or silencing of genes by methylation that are passed on from one generation to the next without altering nucleotide sequences are called epigenetic reactions. Epigenetic reactions are reversible chromatin (gene and protein) alterations that occur in two forms, methylation and acetylations. With methylation, gene and chromatin structures are not available for transcription by replication enzymes; however, with acetylation of lysine or arginine residues in chromatin (histones), gene sequences are available to replication enzymes and transcription may occur [100,101,102,103,104,109,110,111,112,113,114,115,116]. To help understand and these reactions, as well as epigenetic protocols, the reader is advised to consult the text edited by Tollefsbol [111]. For insight into the many epigenetic reactions involved in inflammation and major depression, the reader is advised to consult references [112,113,114,115,116].

#### 2.3.6. Transfer of Aminopropyl Groups and Physiological Effects

**Figure 6:**
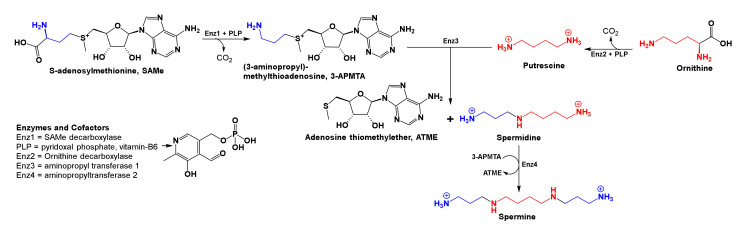
Spermidine and spermine biosyntheses. A high concentration of these amines was found in the hippocampi, and frontal, dorsolateral prefrontal, and temporal cortices of MD patients who completed suicide. This figure outlines the biosyntheses of spermidine and spermine from SAMe and ornithine. Two SAMe molecules donate the aminopropyl groups (blue) and ornithine donates a diaminobutane fragment (red). Reactants, enzymes, cofactors, and byproducts involved in these biosyntheses are shown in this figure [117,120,121,122,123,124].

Along with the methylation and trans-sulfuration reactions mentioned above, SAMe supplies aminopropyl groups to make the polyamines, spermidine, and spermine. Beyond detoxifying free radicals in tissues, polyamines are used in mammalian cells to complex sodium, potassium, and calcium ions in cells and participate in nucleic acid and protein synthesis (Figure 6) [117,118,119,120]. Reports showed that depressed suicide completers had high concentrations of spermidine and spermine in brain tissue extracted from the frontal, dorsolateral prefrontal, and temporal cortices as well as hippocampi. Consequently, a spermidine/spermine *N*-acetyl transferase-1 gene defect is proposed. This enzyme (spermidine/spermine-*N*-acetyl transferase-1) is involved in the breakdown and clearing of polyamines from cells. However, the concentrations of SAMe involved, and how decarboxylation of SAMe in neural tissue causes spermidine/spermine concentrations to become abnormally high in the CNS, are unknown [121,122,123]. This provides another reason why SAMe may not be prescribed to patients who can experience mania; instead, high doses of biotin (100 mg 3x/day) may be prescribed to Alzheimer’s, and traumatic brain injury patients [108,124].

Furthermore, it was interesting to note that the enzyme arginine:glycine amidinotransferase (EC 2.1.4.1) converts arginine and glycine to ornithine and guanidino acetate (or carnitine), and the enzyme arginine decarboxylase (EC 4.1.1.19) forms agmatine ((4-aminobutyl)-guanidine) upon decarboxylation of arginine, which then proceeds to diamino butane (putrescine) for use in polyamine synthesis (Figure 6). Curiously, both carnitine and agmatine are natural antidepressants that appear to be as efficacious as ketamine, a drug which in future could be used as an antidepressant in clinics [125,126].

#### 2.3.7. Inflammation Reduction and Remarks as They Pertain to This Case

SAMe is used in clinics in many countries to reduce steatohepatitis. Experimentally, it was found to increase glutathione levels in rats with inflamed nonalcoholic fatty livers (NAFL) also called nonalcoholic steatohepatitis (NASH). When rats with NAFL/NASH were treated with SAMe, they showed a significant lowering of serum: albumin, and alanine and aspartic transaminases, which are released from destroyed (necroptotic) liver cells. Necroptosis of cells increase proinflammatory cytokines that fuel inflammation and cause bystander tissue damage in addition to summoning neutrophils and macrophages to the site of inflammation. In addition, the dying cells help raise levels of matrix metallo proteases that encourage scar formation, and protein fibers, which form scars in damaged tissues [83,127,128,129,130,131].

Given this background, and that the patient has an enlarged fatty liver (steatohepatitis), uses many medications [1,2], and shows no physiological contraindications for using SAMe, a clear case was made for him to use SAMe cautiously to reduce liver inflammation and major depression. After using SAMe for about six months (with his medications and routines), his annual liver enzyme values were within normal ranges and remain so to date.

### 2.4. Suggested Roles of Vitamin D_3_ for Reducing Inflammation and MD

Discussions on this essential fat-soluble compound with far-reaching physiological effects will be per the following outline:

2.4.1. Vit-D3 precursors and epimers
2.4.2. Bioconversions in sunlight
2.4.3. Dietary supplement, transport, bioconversions in the liver and kidneys, storage, and release
2.4.4. Diffusion through membranes and receptors for vit-D3
2.4.5. Vit-D3 concentrations in health and disease
2.4.6. Catabolism and excretion
2.4.7. Remarks on vit-D3 pertinent to this case

#### 2.4.1. Vit-D3 Precursors and Epimers

**Figure 7:**
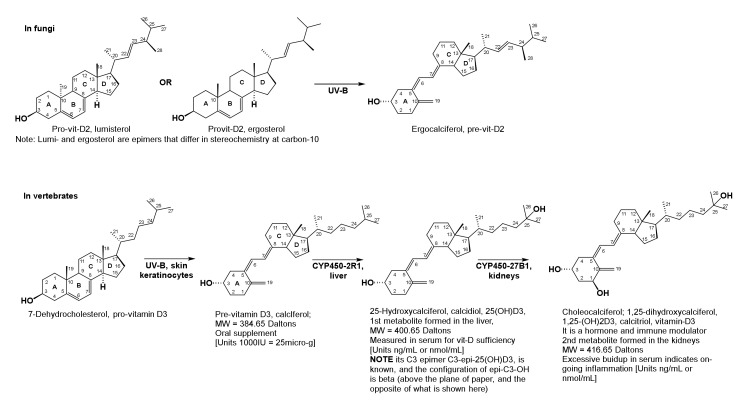
Structures of Vitamins D_2_ and D_3_. Metabolism of vitamin D_3_ in vertebrates. Fungi (mushrooms) produce lumisterol and ergosterol, which is converted to ergocalciferol by UV-B in sunlight (~290–310 nm). Vitamin D_1_ is a mixture of fungal sterols and ergocalciferol (or pre-vitamin D_2_). Structural differences among D-vitamins appear in their side chains, beyond carbon-17 (C17). For example, D_2_-vitamins have a C22-C23 double bond, and a methyl group at C24; and vitamin D_3_ structures do not have these features. Vertebrates produce pro-vitamin D_3_ (or 7-dehydrocholesterol). In sunlight, pro-vitamin D_3_ is converted to pre-vitamin D_3_ or calciferol. In the liver, calciferol is converted to calcidiol, 25(OH)D_3_, which in turn is converted to choleocalciferol or 1,25(OH)_2_D_3_ in the kidneys. Both conversions are caused by different cytochrome P450 mitochondrial enzymes, as shown in the figure. Transport of vitamin-D precursors from the skin or gut to the liver and kidneys is affected by albumins, and α-globulins, respectively. Choleocalciferol is vitamin D_3_, which is both a hormone and immune modulator. Optimal serum levels of 25(OH)D_3_ > 20–30 ng/mL, denote good health. Increased serum 1,25(OH)_2_D_3_ denotes depletion of 25(OH)D_3_ in inflammatory illnesses and infections (see text). Optimum doses for dietary vitamin D supplementation for all age groups and both sexes are not known. C-3-epi-25OHD_3_ is also known; however, not much is known about its physiological chemistry in humans [132,133,134,135,136,137,138,139,140,141].

Pro-D-vitamins are steroid alcohols with four intact steroid rings, A, B, C, and D (Figure 7). Pre-D-vitamins are secosterols wherein the 9–10 bond closing the B-ring is missing, and the A, C, and D rings are intact. The prefix seco indicates that a ring forming bond is missing in a multi-ring molecule. Vitamin D_1_ is a mushroom-derived mixture of epimeric lumisterol and ergosterol (pro-vitamins), as well as the pre-vitamin D_2_ (also called ergocalciferol). Epimers differ in the arrangement of groups (substituents) about a single carbon, which in the case for vitamins D_2_ (vit-D2) and D_3_ (vit-D3) is at carbon-10 (C-10, Figure 7). In pro-vitamin D_2_ (lumisterol), the methyl substituent is behind the plane of paper, and, in pro-vitamin D_3_ (7-dehydrocholesterol), it is above the plane. A naturally occurring C-3-epimer of vitamin D_3_ called C-3-epi-25OHD_3_ is also known, and enzymes for alpha and beta C-3 epimer formation were identified in the liver and kidneys. The term alpha here implies that the substituent hydroxyl group at C-3 is behind the plane, and, in the beta epimer, the hydroxyl group at C-3 is above the plane. Furthermore, while the physiological role and catabolic fate of the C-3-β-epimer is akin to the α-C-3 compound (vit-D3), not much else is known about the physiological chemistry of C-3-epi-vit-D3 (or the C-3-vit-D3-β-epimer).

#### 2.4.2. Bioconversions in Sunlight

Pro-vitamin D_3_ is 7-dehydrocholesterol. It is converted by UV-B radiation (~290–310 nm) in sunlight to the pre-vitamin D_3_, in skin keratinocytes of vertebrates. Phytoplankton (=alga and dinoflagellates) also produce pre-vitamin D_3_ from the same precursor 7-dehydrocholesterol, and are consumed by small pelagic fish (ocean going smelt, bait, etc.). Following predation, larger fatty fish (tuna, shark, swordfish, cod, etc.) accumulate the pre-vitamin in fatty organs (skin, liver, and muscles) and are rich sources of pre-vitamin D_3_. Regardless of the plant or animal species within which the sterols and secosteroids originate, exposure of pro-D-vitamins to sunlight converts them to pre-D-vitamins [132,133,134].

**Figure 8:**
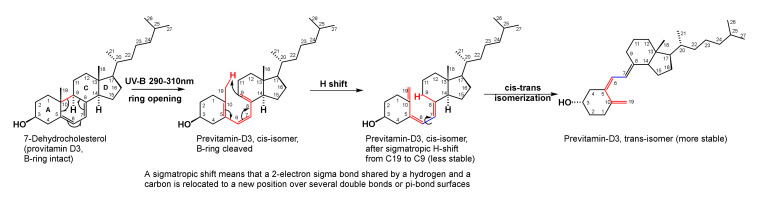
Conversion of Pro-vitamins-D to Pre-vitamins-D. Conversion of a pro-vitamin D to a pre-vitamin D involves opening of the B-ring, a sigmatropic hydrogen shift from C-19 to C9, and a *cis* to *trans* isomerization. The latter isomer is more stable [132,133,134,135].

UV-B induced pro- to pre-D-vitamin conversion involves three successive rearrangements (Figure 8): B-ring opening; a sigmatropic hydrogen (H) shift, and a *cis* to *trans* isomerization because the trans-conformation is thermodynamically more stable. Sigmatropic hydrogen shift in organic chemistry means that a single 2-electron sigma (means covalent) bond shared by a hydrogen and a carbon is relocated to a new position over several pi-bond surfaces, e.g., the hydrogen from carbon 19 is shifted to carbon 9 over multiple double (pi) bonds, shown in Figure 8. Bonds where changes occur are colored red.

Structural differences between vitamins D_2_, D_3_, and their pro- and pre-antecedents appear in the side chain beyond carbon-17 (C17). In vit-D2, carbons C22 and C23 are doubly bonded, and C24 bears a methyl substituent. In vit-D3, these structural characteristics do not exist (Figure 7) [132,133,134,135].

#### 2.4.3. Dietary Supplement, Transport, Bioconversions in the Liver and Kidneys, Storage, and Release

In Western cultures, OTC dietary supplements, dairy products, cereals, and fruit juices are fortified with industrially produced pre-vitamins D_2_ and D_3_ because, in the 20th century, vit-D was discovered to stimulate bone health by enabling intestinal absorption of calcium, phosphate, magnesium, iron, and zinc ions. Both vitamins D_2_ and D_3_ are proposed to participate in the same physiological reactions; however, less is known about the clinical biochemistry of vitamin D_2_ in humans, which only enters the body upon consumption of mushrooms and/or foods fortified with vitamin D_2_. Since vitamin D_3_ is selectively administered and monitored in the clinics, our discussions herein will focus on vit-D3 as they pertain to subduing inflammation and major depression [142,143,144,145].

Upon introduction into the body through diet, or via synthesis from 7-dehydrocholesterol in skin keratinocytes, pre-vitamin D_3_ is carried to the liver by serum albumins, wherein hydroxylation at carbon-25 (C25) with cytochrome P450-2R1, converts pre-vit-D3 to calcidiol, 25(OH)D_3_. Thereafter, calcidiol is carried by serum α-globulins into the kidneys where it is converted to calcitriol (1,25(OH)_2_D_3_), by cytochrome P450-27B1, see Figure 7 [131,132,133,134,135,142,143,144,145].

Enzyme cytochrome P450-27B1, is also called 25-hydroxyvitamin-D-1-α-hydroxylase. It arises from a pleotropic gene, which means that the single gene generates a protein that has many different physiological functions within the same organism. For example, in peripheral immune cells and in CNS neuroglia, cytochrome P450-27B1 produces vitamin D_3_; and the same cytochrome enzyme enables the maturation of monocytes to macrophages and dendritic cells [136,142,143,144,145].

Calcidiol and calcitriol are stored in fatty tissues for long periods, and practically every cell in the body contains vit-D receptors (VDRs) that bind calcitriol. Post synthesis, calcitriol or 1,25(OH)_2_D_3_ is released into the serum as the active form of vit-D3, which is both a hormone and an immune modulator. Figure 7 summarizes the above reactions involved in vit-D3 synthesis from the pro-vitamin 7-dehydrocholesterol, and shows how calcidiol and calcitriol levels are monitored in human health and disease. In addition, it explains the structure of epi-C3-25(OH)D_3_ [132,133,134,135,142,143,144,145].

#### 2.4.4. Diffusion through Membranes and Receptors for Vit-D3

Vit-D3 is a small fat soluble molecule that readily diffuses through cell membranes and the BBB due to its lipophilicity, low ionizability and molecular weight. Receptors for vit-D3 that affect cytoplasmic signaling responses are called cell membrane associated rapid response steroid binding (MARRS) receptors and these are chaperone proteins; these are involved with Ca^2+^ uptake, protein kinase activities, and the like. Vit-D receptors (VDRs) within the cytoplasm, also called hormone response elements (HREs), dimerize with cytoplasmic retinoic acid receptors (RXRs). Thereafter, they (VDR-RXR dimers) carry calcitriol into the nucleus to influence epigenomic and genomic responses by binding and promoting histone acetylation which exposes and enables downstream DNA transcription into mRNA, and subsequent translation to protein [132,133,134,135,136,142,143,144,145].

To provide a gene-vitamin-D3 connection to multiple sclerosis, it was shown that vitamin D_3_ interacts with, and modulates, HLA-DRB1*1501, an allele on chromosome six that is proposed to be associated with the risk for developing MS. However, the exact chromatin (histones, DNA, and RNA) fragments influenced by vitamin D_3_ to subdue inflammation, MS, and MD are thus-far unknown, and are topics of current investigations [146,147,148].

#### 2.4.5. Vit-D3 Concentrations in Health and Disease

It is estimated that healthy individuals exposed to about 12 min of mid-day sunlight in US mid-latitude states could generate about 3000 IU of vit-D3 [132,133,134,135,142,143,144]. IU stands for international units, and one thousand IU of vitamin D_3_ contains about 25 μg of the vitamin (correspondingly 3000 IU = 75 μg). In clinics, serum calcidiol and calcitriol concentrations are measured by tandem high performance liquid chromatography and mass spectrometry (HPLC, MS), and they represent an individual’s health status. However, concentration ranges for 25(OH)D_3_ that help discern healthy, from at risk, and sick individuals by sex and age are unknown [144,145]. The combination of low 25(OH)D_3_ and high 1,25(OH)_2_D_3_ serum values is used to denote poor bone and immune health, Figure 7. Vit-D3 deficiencies are proposed to cause:

- Skeletal maladies such as osteomalacia (bone softening), osteoporosis (bone brittleness), and rickets (bone deformation) [147,148,149,150,151];
- Inflammatory autoimmune diseases, viz-a-vie, rheumatoid arthritis, systemic lupus erythematosus, multiple sclerosis, diabetes mellitus, and cancers [136,146,147,148,149,150,151],
- Increased susceptibility to viral, bacterial, and fungal infections [137,138,152,153,154]; and
- MD, which can lead to suicide [139,140,141].

In their joint Sweden-USA 2014 study, investigators reported that the mean value (+/−SD, standard deviation) of vitamin D_3_ among:

- Suicide attempters was 47 +/− 20 nmol/L (=19 +/− 8 ng/mL, *n* = 59 subjects);
- Non-suicidal depressed patients showed 62 +/− 27 nmol/L (=25 +/− 11 ng/mL, *n* = 17 subjects); and
- Healthy euthymic individuals showed 65 +/− 26 nmol/L (=26 +/− 10 ng/mL, *n* = 14 subjects) [140,141].

In addition, many in the literature have suggested a rule of thumb: A value less than (<) 50 nmol/L = 20 ng/mL is considered deficient in vitamin D_3_; and serum values < 25 nmol/L = 10 ng/mL is seriously deficient [132,133,134,135,142,155].

While prolonged excesses of vit-D3 are proposed to lead to increased calcium deposition in bones and soft tissues, as seen in patients with renal malfunction, protein urea, and metastatic cancers, Holick noted that 10,000 IU/day (=2500 μg or 2.5 mg) vit-D3 supplementation did not result in toxicity. At present, we do not know the optimal doses for vitamin-D_3_ supplementation sorted by age, sex, and health status. However, recent reports from Hollis and others show that iatrogenic doses >500,000 IU (=12,500 μg or 12.5 mg) are toxic to infants and adults, and prolonged use of large doses of vitamin D_3_, are proposed to increase risk of bone fracture among the elderly. Symptoms of acute toxicity (doses exceeding 1,000,000 IU) include, nausea, emesis, pancreatitis, and kidney failure, and symptoms improved upon intravenous administration of fluid, calcitonin, and diuretics [156,157,158,159,160].

Numerous studies have shown that supplementation of the vitamin reduces symptoms of asthma [161,162], diabetes [163], and pruritus [164]. In addition, vit-D3 promotes wound healing by suppressing cytokines (IL’s-1, IL-17, TNF-α, TGF-β, and IFN-γ) that fuel both inflammation and major depression. Furthermore, recently in experiments with mouse bone marrow-derived macrophages and with mice in vivo, it was shown that vitamin D suppresses the NLRP3 inflammasome response to inflammatory stimuli (bacterial lipopolysaccharide, LPS, and alum) upon binding of the vitamin-D receptor (VDR) to the NLRP3 inflammasome, and inhibiting IL-1α secretion [165,166,167,168,169].

With regard to bone, immune, and neurological health, hormones calcitriol (1,25(OH)_2_D_3_, parathyroid hormone (PTH), and calcitonin are known to act in concert to manage calcium (Ca^2+^) and monohydrogen phosphate (HPO_4_^2−^) homeostasis that enable bone mineralization, and metabolic, immune, and neurological functioning [170].

In patients with Crohn’s disease, it was found that, during active phases, when bowel inflammation and pain were high, patients showed lower levels of 25(OH)D_3_. The extent of 25(OH)D_3_ nadir (low point) in serum conversely matched increased disease severity, high patient-reported Harvey Bradshaw scores, high gene expression for vitamin D receptors (VDRs), and higher numbers of peripheral T cells in inflammatory phases. During remission from Crohn’s disease, the opposite relationships were found. This suggested that increase in VDRs during inflammation corresponded with depletion of 25(OH)D_3_ and an increase in 1,25(OH)_2_D_3_ concentration in patients’ sera (Figure 7). Likewise, during remission or upon treatment with the anti-inflammatory agent infliximab; decreased inflammation corresponded with increased serum levels of 25(OH)D_3_ and decreased levels of the hormone 1,25(OH)_2_D_3_ and its VDRs, and lower Harvey Bradshaw scores [171,172]. Furthermore, in a study involving patients with hyperparathyroidism, excess parathyroid hormone (PTH) in the serum led to a severe deficiency of 25(OH)D_3_, and corresponded with an increase in depressive symptoms as indicated by high self-reported Beck Depression Inventory Scores [170,171,172,173,174,175].

#### 2.4.6. Catabolism and Excretion

**Figure 9:**
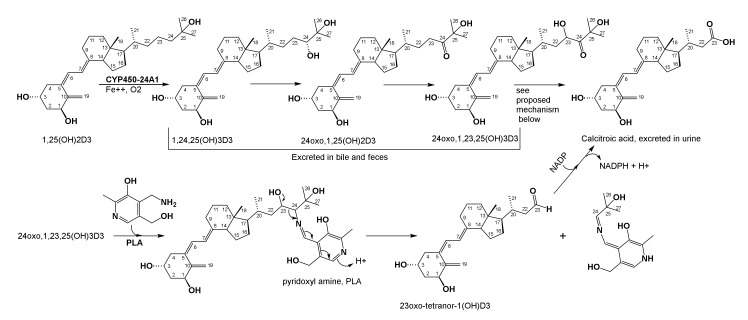
Proposed mechanisms for breakdown and excretion of vitamin D_3_. Except for calcitroic acid which is excreted in urine, all other poly-hydroxy-vitamin-D_3_ derivatives, [1,25(OH)_2_D_3_, 1,24,25(OH)_2_D_3_, 24oxo,1,25(OH)_2_D_3_, and 1,23,25(OH)_2_D_3_] are excreted in bile [176,177,178]. Cleavage of the side chain at C23 is proposed to involve pyridoxyl amine, PLA.

Breakdown of 1,25(OH)_2_D_3_ occurs by successive oxidations at carbons 23 and 24 by the mitochondrial enzyme, 1,25-dihydroxyvitamin-D_3_-24-hydroxylase or cytochrome P450 24A1, which is a multicatalytic mitochondrial enzyme that hydroxylates both C23 and C24. Almost all vit-D3 generated by synthesis in skin or consumed by diet is excreted in the bile and feces as fat soluble poly hydroxyl vitamin D3 derivatives formed by cytochrome P450 oxidations. Continued side chain oxidations and cleavage result in the water soluble calcitroic acid, which comprises 2–4% of all D3 breakdown products excreted in urine (Figure 9) [176,177,178].

In 2015, this patient (primary author, NS) had to stop using DMF because with its use he developed leukocytopenia. Given precedents summarized above regarding the anticipated benefits an MS patient could derive by consuming vit-D3, it was suggested to him that he consume vit-D3, and continue with his medications for other illnesses, as well as his exercise and meditation routines. Several months later, he realized that his BDI scores had reduced from high 20s to mid 30s to between 6 and 18, and thereafter, for ~17 months, the patient enjoyed a period of productivity and euthymia. Later, he experienced difficulties with his brain tumor, sinus infections, and CN6 palsy, and to date has poor vision; however, no new MS-related lesions have appeared in his MRIs; and bupropion supplemented with SAMe and vitamin D_3_ seem to maintain equanimity.

## 3. Conclusions and Hope for the Future

In this paper, we’ve highlighted the neuroimmune aspects of four neuroprotectants DMF, bupropion, SAMe, and vitamin D_3_, which, when used by this MS patient after all treatments were exhausted, improved his well-being and productivity [1,2]. Weeks after he started with DMF, he transited from walking with a walker to walking with crutches because it appeared to have subdued his MS-related intercostal cramping, and reduced the frequency and intensity of his asthma and urticaria symptoms. However, within one year of use, it caused leukocytopenia and was withdrawn. Bupropion (above all previously used antidepressants) further reduced agitation and frustration with MS related function losses and depression, improved the patient’s ability to deal with temperature changes and walk short distances in hot weather, and enabled him to lose much weight he had gained with steroid use (for MS, lumbar pain, and asthma). Furthermore, additional benefits were realized when SAMe and vit-D3 were introduced as adjuvants to bupropion. Within months of introducing this cocktail, his BDI scores went from upper 20s to 30s to low teens and single digits. In addition, benefits of this combination continue to date, despite intervening difficulties with his brain tumor and bitemporal vision loss, then sphenoid sinus infections causing CN6 palsy and diplopia, and their treatments [1,2]. So, herein, we’ve summarized current neuroimmune mechanisms by which:

- DMF when orally consumed, hydrolyses to MMF, which then reacts with glutathione to form the glutathione-succinate complex. This complex suppresses the NLRP3 inflammasome, as noticed by: reduction in inflammation; decreased serum levels of inflammatory cytokines; and upregulated serum concentrations of antioxidants. Furthermore, DMF was reported to reduce excessive build-up of oxidized tryptophan metabolites (kynurenic and quinolinic acids, as well as 3-hydroxykynurenine), which, if left to accrue unchecked, can cause excitotoxicity and tissue losses in the CNS and PNS, dysregulate the HPA axis, and disrupt neurotransmitter, endocrine, and immune functioning.
- Bupropion upon oral consumption metabolizes to hydroxybupropion that lasts in serum longer than bupropion, and functions as a stimulant and antidepressant. It is known to: maintain adequate serum and CNS neurotransmitter concentrations to help alertness, learning, cognitive functioning, memory, and mood; improve endurance during strenuous activities; increase libido in depressed patients; reduce inflammation and the perception of pain; decrease the need to smoke and overeat; and balance innate and adaptive immune functions when fighting excessive stress. In addition, we discussed why bupropion as well as SAMe may not be prescribed to patients who experience mania, or tend to get excessively agitated when in pain. To facilitate readers’ understanding of the psychoneuroimmunology of bupropion, we enpassant (in passing) summarized physiology concepts of neurotransmitters in Box 1 to ease following our discussions on bupropion.
- Regarding the neuroimmune roles of SAMe in subduing inflammation and MD, we discussed its biosynthesis, chiral aspects affecting its biological activity versus toxicity, and temperature and ionic conditions affecting its stability. Thereafter, we discussed its roles in: one carbon metabolism; its own build up and catabolism in different biological cycles; the biosynthesis of methionine, glutathione, and cysteine, that, in turn, reduce free radical oxidative damage in CNS and PNS; and epigenetic reactions that are implicated in relieving inflammation and major depression. We introduced additional concept summaries in Box 2, so readers may compare the roles of SAMe with other one carbon cofactors; the fragments they transfer; and how they are described to reduce inflammation and major depression. Still further, we discussed the physiological chemistry of spermidine and spermine as they are reported to accumulate in CNS tissues of patients who completed suicide.
- Our discussions on the neuroimmune chemistry of vit-D3 involved: its biosynthesis, dosage, structural changes in pro and pre-vitamin forms that produce the active hormone and immune modulator (calcidiol); and its serum concentrations that are reported to subdue inflammation, and excessive buildup of its metabolite calcitriol which specifies on-going inflammation or neuroimmune diseases. In addition, we discussed mechanisms by which vit-D3 is reported to subdue inflammation in patients with MS, MD, asthma, type-2 diabetes, and Chron’s, bone, and other inflammatory diseases by blocking the inflammasome NLRP3 in forming and releasing inflammatory cytokines. Our discussions on vit-D3 ended by showing the successive side chain oxidations that catabolize the vitamin for excretion.

Major depression in chronically ill patients could be due to stresses of inflammatory illnesses, and changes in life circumstances (vis-à-vis, inability to function the way a person once did, job loss, sudden social changes, and not knowing how to emerge from the morass). Consequently, patients with chronic medical diagnoses require many medical specialists who can manage patient needs. This patient is fortunate because family members, friends, former educators, colleagues, and care team members were/are proactive, who helped/help him find and discuss literature to support appropriate decisions that explained his conditions, methods to effect relief, and created in him an unwavering desire to improve his status and become functional once again. In addition, he was encouraged to maintain records of his and care-taker’s observations of treatments and the risks and benefits they afforded. This may be difficult for other patients. However, with coaching from physicians and their assistants, patients could be motivated to consult the literature, visualize the changes they desire, and attain their health goals without giving in to pain and disability. Our impetus to write these papers is with the hope that, in the future, patients and physicians will be encouraged to write their own case reports and explain the science behind their thinking and treatment choices, so a wide body of literature is available in peer-reviewed venues so chronically ill patients and physicians can learn from these examples, individualize their care, and feel productive and euthymic despite illness.
